# Infusing creativity into a college course: a pilot study

**DOI:** 10.3389/fpsyg.2026.1768412

**Published:** 2026-05-14

**Authors:** Jean E. Pretz

**Affiliations:** Department of Psychology, Elizabethtown College, Elizabethtown, PA, United States

**Keywords:** academic achievement, assessment, creativity, higher education, mindset, pedagogy, self-efficacy

## Abstract

Creativity is an essential skill which has been undervalued in higher education. Creativity is increasingly important to both educators and employers, yet several obstacles challenge its inclusion in college curriculum, including student and faculty misconceptions, and lack of models of how to integrate creativity into college courses. This pilot study designed and evaluated a creativity-infused version of a traditional college course, implementing numerous evidence-based best practices in fostering creativity from the psychological literature on the subject. Details on creativity activities, assignments, and assessments are described, including the “Minute Meme,” the “Innovative Illustration” assignment, the “Creative Correction” assignment, and the “Creative Paper Project.” A small pilot study showed preliminary evidence for the effectiveness of the intervention for students' self-efficacy and growth mindset about creativity. Students also provided positive reviews of the intervention activities. Guidance on how to adapt these types of activities into courses in any discipline is provided, and suggestions for specific future research studies to further evaluate the effectiveness of this approach are discussed.

## 1 Introduction and objective

Creativity is an essential 21st century skill in a rapidly changing world where adaptability and problem solving are increasingly important. Both educators (Egan et al., 2017; Soule and Warrick, 2015) and employers desire creativity (Finley, 2021). Yet creativity has been historically undervalued in higher education (Sternberg, 2010). A recent meta-analysis revealed minimal research on creativity in higher education, not even a common definition of the term (Fox and Smith, 2024). Students lack confidence in their abilities (Kaufman and Beghetto, 2013), hold misconceptions about the construct (Wieth and Francis, 2018), and instructors have few models of how to foster creative thinking in the classroom. The objective of this study was to report details of an intervention that infused creativity into a college course and to provide a “proof of concept” and preliminary pilot data supporting its potential for further study.

### 1.1 Creativity in higher education

Higher education has emphasized critical thinking over creative thinking (Halpern, 2007). Yet Sternberg (1999) places creativity on par with analytical thinking and the ability to apply and use practical thinking as equally important components of human intelligence. Current college students, members of “Gen Z,” have been characterized as less creative than older generations (Twenge, 2013). National and state standards can interfere with efforts to develop students' creativity. Student and teacher beliefs about creativity can pose an obstacle. Students often have a narrow definition of creativity as relevant only to the arts, limiting their perception of their own creativity (Wieth and Francis, 2018). Some teachers fail to distinguish creativity from academic achievement (Kagan et al., 2025), and teachers differ in their beliefs about whether creativity is innate or can be fostered (Katz-Buonincontro et al., 2020).

Creativity has been found to correlate with academic success. In a study of college applicants, we found a weak but significant relationship between creativity and standardized test scores and high school rank (Pretz and Kaufman, 2015). A meta-analysis of 120 studies of students from elementary school to college found a weak to moderate positive relationship between creativity and academic achievement (Gajda et al., 2017). Past research on creativity in college students has shown that its relationship to academic success varies depending on year in college. Among first-year students, creativity was related to grades, but this relationship was not found among fourth-year students (Pretz and Kuczma, 2025). When standardized test scores and critical thinking scores were included with creativity as predictors of college grades, creativity had no significant unique relationship to academic achievement (Pretz and Kuczma, 2025).

The relationship between creativity and grades also varies by discipline. Self-perceptions of creativity were positively related to college GPA, even controlling for high school grades. However, this was only true for students majoring in Arts, Humanities, and Social Sciences fields (Pretz and Kuczma, 2025). In fact, for science majors, a trend in the opposite direction was found. A study of engineering students also found a significant inverse relationship between self-perceived creativity and academic performance. Science students who had higher creative self-efficacy had lower grades (Atwood and Pretz, 2016). Kile et al. (2016) found that 1st-year engineering students with the highest fluency scores on a creativity task had significantly lower grades than their less creative peers.

Even students who have high creative self-efficacy and want to be creative may avoid doing so. Beghetto (2014) documented a phenomenon called “creative mortification” which reflects student embarrassment or shame at taking a creative risk that is not well-received by peers or teachers.

### 1.2 Fostering creativity in the classroom

Brauer et al. (2025) provide a comprehensive and in-depth overview of teaching practices that foster creativity. In their meta-creativity model, they argue that creativity can be fostered by focusing on these six dimensions: affect, cognition, behavior, metacognition, creativity-in-action (finding meaning in the process), and uncertainty. Specific strategies to foster creativity in the classroom involve creating a supportive environment. This includes explicit instruction on what creativity is, nurturing students' intrinsic motivation, conveying the value of creativity, and providing feedback on creative work.

Research has shown that explicit instructions are often necessary to elicit creativity in a classroom setting. The default for students tends to be a search for a single “correct” response, in part because that is what is frequently an appropriate strategy in school. Eisenberger et al. (1998) found that when promised a reward, elementary-age children produced work that was less original and more elaborate, but when the reward was combined with clear instructions to be creative, it resulted in higher rates of creativity. A meta-analysis by Acar et al. (2020) confirmed that creativity on divergent thinking tasks was higher when instructions emphasized creativity and quality of work rather than simply quantity of responses. Clearly instructions that highlight the value of creativity are important, but there is one caveat. Past research has shown that providing specific examples of creative work can lead students to create work that conforms to elements of the examples, reducing their creativity (Marsh et al., 1996). Instructors who share examples with their students should provide numerous diverse examples to avoid this pitfall.

Intrinsic motivation is a key component to fostering creativity. When a student is personally invested in their work because of their own intrinsic interest in the topic, they will do higher quality and more creative work. In contrast, extrinsic motivators such as high-stakes grades have been shown to limit creativity (Hennessey, 2010). Student motivation to be creative can be enhanced in numerous ways including by encouraging collaboration, risk-taking, and accepting that mistakes are inevitable (Hennessey, 2015).

Past research on fostering creativity among college students recommended explicitly requiring creative responses, employing open-ended assignments for which students can develop several possible ideas to evaluate before focusing on a single option, and using a target audience as a way to increase creativity of ideas (Wieth and Francis, 2018). Another study showed that students felt that their creativity was fostered in courses where they could brainstorm and work on open-ended projects that included scaffolding and reflection, and courses in which they could learn from the work of others and receive ample instructor feedback (Daly et al., 2016).

## 2 Pedagogical framework

### 2.1 Teaching about, for, and with creativity

Beghetto's (2017) framework proposes that creativity be integrated into the classroom by teaching *about* creativity, teaching *for* creativity and teaching *with* creativity. This approach can be employed in any course in any discipline. Teaching *about* creativity involves helping students recognize what creativity is. This may also include discussion of examples of creative contributions in particular discipline, emphasizing factors that influence creativity, both positively and negatively. Teaching *for* creativity focuses on fostering students' creative abilities. This can involve using programs that are intended to train for creativity directly or fostering student creativity through activities in any discipline. Teaching *with* creativity involves the instructor using a creative approach to deliver course material. In this way, the instructor models creativity including risk-taking, learning from failure, expressing openness to new ideas, using flexible thinking, and seeking complexity.

### 2.2 Best practices in teaching for creativity in college

Guided by Beghetto's (2017) framework and insights from Brauer et al. (2025) as well as findings from past research on creativity, a list of best practices were developed to implement in a creativity-infused intervention.

- Overcome students' misconceptions about creativity by conveying the nature of creativity
- Model creativity through teaching itself
- Encourage risk-taking, promote psychological safety
- Harness students' intrinsic motivation through choice in assignments
- Share numerous diverse examples of highly creative student work as inspiration
- Reduce extrinsic motivators through low-stakes activities
- Build creative self-efficacy through practice and feedback
- Value holistic student abilities by explicitly assessing creativity as part of course grade

### 2.3 Hypotheses

In order to give students the opportunity to practice creative thinking and receive feedback on their work, we implemented a creativity-infused version of a traditional course. We predicted that those in the intervention course would show 1) higher levels of creative performance as compared to students in the traditional course, 2) greater creative self-efficacyand 3) a stronger growth mindset about creativity over the course of the semester (from pre-test to mid-term to the end of the course), while 4) performing similarly on other memory-based and analytical-based components of the course as compared to students in the traditional course. We also predicted that 5) performance on creative assessments would be distinct from traditional assessments.

## 3 Learning environment

### 3.1 Participants

Participants were 46 3rd- and 4th-year undergraduate students enrolled in a 300-level course in Cognitive Psychology at a small college in the Mid-Atlantic region of the US. Two sections taught by the same instructor were included in the study, one taught in Fall 2023 (control group, *N* = 24) and one taught in Fall 2024 (intervention group, *N* = 22). Of the 25 students in the Fall 2024 section, 22 agreed to participate. Students were largely of traditional college age (*M* = 21.00, *SD* = 3.34), with 16 female and 6 male participants, all of whom reported White race/ethnicity. The study was approved by the Institutional Review Board, and all enrolled students were invited to participate. All class members received the intervention as part of the course delivery, but only those who voluntarily consented to participate in the research completed the self-report measures. In order to avoid coercion, the instructor was unaware of which class members actually participated in the study and did not see responses to the surveys until after final grades were submitted at the end of the semester. Grades from all 24 students in the Fall 2023 section were obtained archivally.

### 3.2 Materials

Students in both groups completed the same traditional assessments and the Creative Paper Project. The intervention group additionally completed self-report measures of creativity during the 1st week of class, at midterm, and during the last week of the course. Students provided feedback about the intervention itself at the end of the semester.

#### 3.2.1 Traditional assessments

Scores on three exams were averaged to create an overall exam score for the course. Each exam included multiple choice and short essay questions on course material that required critical thinking and application.

All students wrote an analytical paper on a topic of their choice related to cognitive psychology. This 10–12 page paper required students to form a thesis and provide a review of the literature based on original empirical research articles. The paper was scored using a rubric that evaluated quality of summary, synthesis, and analysis, as well as quality of writing.

#### 3.2.2 Creative assessments

Students in both sections created a Creative Paper Project based on the topic of their analytical paper. Students chose a creative medium, selected key ideas from their paper to highlight, created the project, and shared it with the class via an informal presentation. While introducing the assignment to both sections, the instructor showed students the rubric and emphasized the importance of creativity for success on this project. Students were shown several diverse examples of highly creative work from past students including a crocheted neuron, a pop-up children's book about parts of the brain, a backpack full of rocks representing fixed mindset, and a video criticizing the effectiveness of “Brain Games” for enhancing cognition using a political ad style.

Projects were assessed using a rubric that assessed five dimensions on a 4-point scale (see Table 1). The weight of each component toward the total creative project grade is included in parentheses. Creativity (35%), accuracy (15%), depth of understanding (15%), complexity/effort (25%), and quality of explanation (10%). This rubric emphasizes creativity and the effort put into making it a complex integration of information into the creative medium. It intentionally does not evaluate aesthetic quality of the work, though this is generally reflected in the complexity/effort component of the rubric.

**Table 1:** Rubric for assessment of creative paper projects.

Dimensions	Lacking	Emerging	Mastering	Exceeding	Score
Provides an accurate representation of literature in the field	Represents incorrect or very incomplete information (9 points)	Represents mostly accurate information, may be incomplete (11 points)	Represents accurate information in the literature (13 points)	Represents clear, accurate, representative information in the literature (15 points)	/15
Reflects deep understanding of a concept/construct/set of evidence	Reflects lack of understanding (9 points)	Reflects some understanding (11 points)	Reflects strong understanding (13 points)	Reflects deep understanding (15 points)	/15
Is creative (original, unusual, unexpected, different, clever, unique)	Not creative, mundane (23 points)	Somewhat creative (26 points)	Creative (30 points)	Highly creative (33 points)	/35
Represents effort, is complex	Represents little effort, one-dimensional (16 points)	Represents some effort, one-dimensional (19 points)	Represents effort, multi-faceted (21 points)	Represents effort, very complex (24 points)	/25
Is explained clearly to class	Unclear, confusing, doesn't get at main point (6.5 points)	Somewhat clear about main point (7.5 points)	Clearly explained project (8.5 points)	Very clearly highlights literature (9.5 points)	/10
Total					/100

The weight of each aspect of the rubric is reflected in the points in the far right column. For example, Creativity received the highest weight with 35 points. Specific point values for each level of each aspect of the rubric are provided in the table. These were calculated roughly based on a scale in which lacking corresponded to 65%, emerging 75%, mastering 85%, and exceeding 95%. Total score for the creative project was recorded out of 100 points. Analyses were conducted using the total score as well as the scores on the individual aspects.

#### 3.2.3 Survey measures of creativity

The intervention group additionally completed a self-report survey during weeks 1, 8, and 15 during the 15-week semester. Creative self-efficacy was measured using Beghetto's (2006) three-item measure, e.g., “I have a good imagination.” Participants responded on a 5-point Likert scale (strongly disagree to strongly agree). Creative mindset was assessed using Karwowski's (2014) 10-item measure, yielding scores on fixed mindset (“Some people are creative, others aren't—and no practice can change it.”) and growth mindsets (“It doesn't matter what creativity level one reveals—you can always increase it.”) based on responses to items using a 5-point Likert scale (definitely not true to definitely true). Students in the traditional course did not provide data on creative self-efficacy or creative mindset.

Student feedback on intervention was obtained using seven items on 5-point Likert scale including statements such as “These activities helped me increase my own creativity,” “I enjoyed working on these activities,” and “I want to do more creative activities like these in the future.”

### 3.3 Procedure

#### 3.3.1 Traditional course delivery

The traditional course structure was primarily interactive lecture. Three exams were given at even intervals during the semester. The analytical paper was due during the second half of the semester, and the Creative Paper Project was due in the last week of the semester. In addition, students worked in groups of three to prepare presentations on applied aspects of the course topics which were presented in class periodically throughout the semester.

#### 3.3.2 Creativity-infused course

The creativity-infused course largely resembled the traditional course in its interactive lecture format. The same exams, analytical paper assignment, and Creative Paper Project were given. The primary changes were (1) the addition of a lecture on the nature of creativity during the 1st week of the course, (2) enhanced lectures designed to model creativity, and 3) creative activities/assignments throughout the semester which replaced the group presentations on applied course topics. Students in the intervention section of the course also completed survey measures during the 1st week (prior to the lecture on creativity), at midterm, and during the last week of the course. Creative activities were designed to be scaffolded, 1st using ungraded, low-stakes, brief activities to allow students to practice creative thinking, then lightly graded group projects in which feedback was provided at multiple steps in the creative process, and concluding with an individual creative project that constituted 10% of the overall course grade.

##### 3.3.2.1 Lecture and discussion on the nature of creativity

The lecture was designed to share key points about the psychological literature on creativity, emphasizing points that challenged misconceptions about the construct. In a 30-min lecture, the instructor explained that creativity reflects ideas that are novel and task appropriate and that creativity occurs in every domain, providing concrete examples such as Michaelangelo's “The Creation of Adam” on the Sistine Chapel, Nobel Prize winner Marie Curie's scientific discoveries, Mark Zuckerberg's development of social media tools, and a car mechanic as a creative problem solver. Students were introduced to Kaufman and Beghetto's (2009) 4-C model of creativity which argues that creativity can occur at any level, from personal insights to everyday creativity to professional contributions to eminent works. The instructor also provided information on Sternberg's (1999) view of creativity as a component of intelligence, emphasizing the diverse nature of human ability. The class then engaged in a think-pair-share activity about how the literature confirmed or challenged their personal views of creativity. Through large group discussion, the instructor reiterated these key points and further expanded on the benefits of creativity for personal wellbeing and to society.

##### 3.3.2.2 Modeling creativity through teaching

The instructor included elements of creative work in the delivery of course material. For example, in describing Baddeley's model of working memory, an artistic representation was presented, and students were asked to consider the artist's creative choices in representing the various components of the working memory model. Throughout the course, the instructor used psychology-related memes to reinforce key concepts. Class discussion questions were selected to encourage use of creative thinking, e.g., emphasizing “What if” questions such as “What if humans did not have to sleep?” or “What if computers were designed to function more like brains?.”

##### 3.3.2.3 “Minute Meme” activity

One early opportunity for students to exercise their own creativity in the course was an ungraded low-stakes response to a lecture. A variation on the “minute paper” active learning technique (Soetaert, 1998), students were invited to reflect on a key takeaway from the lecture and post a meme or visual about it on a class discussion board. This ungraded assignment was completed during the last few minutes of class and allowed students to express their creativity in a low-stress environment. Class members could appreciate their peers' ideas, and the instructor took the opportunity to reinforce the creativity and risk-taking evident in the student posts. See Figure A1 for sample student work.

##### 3.3.2.4 “Innovative Illustration” assignment

Students completed group projects that were designed to assess creativity as well. The first was an innovative illustration of a course concept in the form of a public service announcement (PSA). Specifically, students created a PSA in response to research on attention showing that multi-tasking is not very effective (e.g., in the context of studying or distracted driving). In this assignment, students worked in groups of three to brainstorm the key points from the course that they wanted to convey in their PSA. Then they brainstormed the possibilities afforded by a creative medium and considered several ways to illustrate the points in the medium. Then they chose what they thought would be the best medium and key points they would convey in the PSA. Notes were submitted to the instructor for feedback followed by a draft which also received feedback prior to final submission. See Figure A2 for sample student work.

##### 3.3.2.5 “Creative Correction” assignment

For another group project, students worked together on a creative correction of a myth or popular misrepresentation of a course concept. Specifically, students analyzed a popular film representation of memory and then prepared a creative project that rewrote aspects of the film to correct them for accuracy. Students worked in groups of three to choose their film, diagnose errors, and brainstorm ways to correct the errors through critique or a creative re-write. The instructor provided feedback on their initial ideas. Students wrote up their analysis documenting at least three errors in the film, using the textbook evidence as support. The instructor graded the analysis prior to the submission of the creative correction portion of the assignment. See Figure A3 for sample student work.

##### 3.3.2.6 “Creative Paper Project” assignment

In the creativity-infused course, each student completed the same Creative Paper Project as students in the traditional course with minor changes. To facilitate diverse and unusual possibilities for creative mediums, the instructor employed a “topic-medium mash up” approach. Students drew a medium (e.g., visual art, skit, song parody, board game, children's book, advice column) at random from a jar and described how their topic could be illustrated in that medium. They submitted their notes on initial ideas based on two randomly selected mediums and one self-generated medium for feedback. In addition to the informal presentation of the project to the class, students also wrote a brief reflection paper explaining their creative process and how their project conveyed key ideas from their paper. See Figure A4 for sample student work.

#### 3.3.3 Creative process, feedback, and assessment

For the creative components of the Innovative Illustration, Creative Correction, and Creative Paper Project assignments, students submitted notes on their ideas using multiple creative mediums. The instructor provided brief feedback on students' initial ideas, emphasizing that the best ideas were the most unexpected, unusual, original, and creative ideas. The instructor also helped students see how the course concepts could be elaborated or more fully expressed via the creative mediums. Then students used the feedback to select one creative medium and make the actual creative project. The final projects were either shared with the class via a discussion board (early on in the course) submitted to the instructor directly. For discussion board submissions (the Innovative Illustration assignment), class members took time during class to practice using the creative project rubric (see Table 1) to identify strengths in their peers' submissions. Through a large group discussion, the instructor highlighted the most creative ideas, reinforcing the value of creativity in the classroom.

In order to protect the internal validity of the quasi-experimental design of this research, peer feedback was not used on the Creative Paper Project assignment, nor was it used for formal evaluation of any creative components of the course. The instructor assessed all student work independently of any group discussions of an assignment using the rubric. The Creative Paper Project which was assigned to students in both sections of the course was evaluated using the same rubric by the same instructor, providing a strong basis for comparison. The key difference in the two groups was that students in the creativity-infused class were required to submit notes on their plans for the project in advance, whereas those in the control condition were merely encouraged to do so voluntarily by reaching out to the instructor informally.

## 4 Results

### 4.1 Impact of creativity-infused course on student creativity

We analyzed quantitative data for graded activities and self-report measures of creativity.

Mean comparisons between total scores on the creative project among students in the creativity infused course (*M* = 93.88, *SD* = 4.89) and those in the traditional section of the course (*M* = 93.91, *SD* = 4.35) revealed no significant difference, *t* (44) = −0.0249, *p* = 0.98. This did not confirm our first hypothesis, but further analyses showed that scores on exams and the analytical paper also did not differ between the sections, showing that the emphasis on creativity in the course did not detract from the students' mastery of course material.

Students in the creativity-infused course increased on both creative self-efficacy and growth mindset about creativity over the course of the semester, confirming hypotheses 2 and 3. From pre-test to midterm to the end of the semester, creative self-efficacy increased, [*F*_(2.30)_ = 3.64, *p* = 0.038, η^2^ = 0.045], with a significant difference between midterm (*M* = 3.60, *SE* = 0.140) and end-of-semester self-efficacy (*M* = 3.87, *SE* = 0.132), *p* = 0.045. Growth mindset also increased across the three time points in the term, [*F*_(2.28)_ = 3.50, *p* = 0.044, η^2^ = 0.046], though *post-hoc* analyses did not reveal significant mean comparisons. These longitudinal data showed significant change over time, but because these data were not available for students in the traditional section of the course, it is not possible to know whether maturation or other confounding variables could have contributed to the changes observed.

Because we expected performance on creativity assessments to reflect strengths in student abilities that were not captured by traditional assessments (hypothesis 4 and 5), we correlated creative project scores with exam scores and analytical paper grades across both sections of the course (*N* = 46). Total creative project scores were non-significantly correlated with exam average (*r* =.254, *p* =.088) and unrelated to scores on the analytical paper (*r* = 0.122, *p* = 0.419), confirming hypothesis 4. Total creative project scores were correlated with course average (*r* = 0.322, *p* = 0.029). Considering specifically the aspects of the rubric for the creative project, the “creativity” aspect of the overall creative project score was also not correlated with exam average (*r* = 0.186, *p* = 0.216), paper scores (*r* = −0.036, *p* = 0.812), or with course average (*r* = 0.189, *p* = 0.208). The only aspect of the creativity project rubric that was related to exams or paper scores was “accuracy.” Accuracy of understanding the course material as reflected in the creative project was moderately related to exam average (*r* =.322, *p* =.029), analytical paper scores (*r* = 0.438, *p* < 0.001), and with course average (*r* = 0.511, *p* < 0.001). The rubric aspect assessing depth of understanding on the creative project was also positively correlated with course average (*r* = 0.373, *p* = 0.011). These results are a first step toward supporting the prediction that creativity would provide a more holistic view of students' abilities. Here we showed that these creativity assignments enable instructors to assess aspects of student success that are not captured by traditional assessments.

### 4.2 Student response to creativity-infused course intervention

End-of-semester feedback from students in the creativity-infused course was positive. Students reported that the creativity activities in the course increased their creativity (*M* = 4.41, *SD* = 0.870), were enjoyable (*M* = 4.24, *SD* = 0.752), were a valuable aspect of the course (*M* = 4.00, *SD* = 0.866). Students reported that they also hoped to do more creative activities in the future (*M* = 3.94, *SD* = 0.899). Two items were less strongly endorsed. Students were in less agreement that the creative activities deepened their understanding of the course material (*M* = 3.76, *SD* = 0.831), and they were less certain that the feedback their received on their creative work was helpful (*M* = 3.71, *SD* = 0.920). Overall, these results are encouraging. Students had a positive impression of these activities and found them worthwhile, enjoyable, and felt that the activities increased their creativity, which was a primary goal of the intervention.

## 5 Discussion of practical implications

### 5.1 Lessons learned

Infusing this course with creativity increased students' beliefs about their own creative potential over the course of one semester. Students enjoyed the experience and even hoped to do more creative work in future courses.

The effort required by the instructor to infuse the course with creativity was comparable to common revisions to class activities, and in this case simply involved replacing existing activities with others that were more creativity focused. One addition was lecture and discussion related to creativity that required about 50 min of class time during the 1st week. This content could easily be integrated into an early lecture on the history of a field, referencing the evolution of ideas in a field and the creative innovations of its leaders.

Creative assignments and activities can be integrated into most any class day (Minute Meme). Innovative Illustration and Creative Correction activities require students to explore a topic in more depth, so they lend themselves well to the most important course topics, applied topics, or those that illustrate a recurring theme in the course. Giving students choices (e.g., specific topic, creative medium) serves to enhance intrinsic motivation and personal investment in the work. The Creative Paper Project assignment could be added to any research paper assignment in a course, giving students an opportunity to share what they have learned through their research with the entire class rather than just the instructor, incentivizing them to do high quality work for a more public audience. Creative projects can be shared more publicly through a scheduled in-person exhibit or virtually through social media or a class website. This is best done later in the semester so that students do not experience the negative impacts of extrinsic motivators until after they have had a chance to grow in creative self-efficacy.

Giving feedback on students' preliminary ideas for their creative work can be straightforward, simply reinforcing the creativity in the students' work or by encouraging students to push themselves to entertain ideas that are truly unexpected and even “wild and crazy.” Evaluation of final creative projects is facilitated by use of the rubric in Table 1. Time spent on feedback can be made more efficient by assigning group projects or relying on peer review.

### 5.2 Implications for higher education

As higher education adapts to a changing student body and responds to financial and political challenges, it is more important than ever that faculty and administrators demonstrate the relevance of programs and curricula. Students must be prepared to make an impact on a rapidly changing environment, and creativity is essential. To nurture creativity in college, faculty and students must overcome misconceptions to develop a broader understanding of creativity. This will allow students to become more aware of their own abilities and for faculty to recognize and reward them. As faculty respond to students' desire for more active and experiential learning, it is easy to bring creativity into those learning experiences. Students can demonstrate learning of course concepts using an “Innovative Illustration” assignment, perhaps in place of a traditional quiz or essay assignment. An analytical assignment can be expanded to include a “Creative Correction” element, having students not only critique ideas but correct them using a creative medium. Any given class session can be concluded with a “Minute Meme” that asks students to reflect on a key takeaway while practicing creative thinking. Students can share their work with classmates or with friends and family outside of the classroom, further reinforcing the relevance of the course content and their work for the course. These activities can be implemented as individual or group assignments, and over time, instructors will amass a collection of creative work from their past students that they can then use as their own “Innovative Illustrations” as they model creativity through their own teaching. Infusing a course with creativity can be energizing for both students and instructors.

## 6 Limitations and future directions

The results of this pilot study are encouraging, but much work is needed to affirm the value of this approach. The study showed higher scores on self-reported aspects of creativity at the end of the creativity-infused course as compared to the beginning, but without self-report data from the traditional course, it is not possible to conclude that the creativity-infused course actually caused the positive effects observed. Future work must employ a true control group for all measures of interest. Another limitation is that the sample was very small and not representative of most college students, and the study was implemented by a single instructor. The infusion was attempted only in one upper-level course in psychology. Future work should infuse courses at multiple levels and in multiple disciplines by multiple instructors to observe its generalizability and effectiveness. The findings should be considered preliminary until a more rigorous test with a larger sample can be conducted. Students of all majors and levels of experience should be included as past work has shown that class year and major field of study affect self-perceived creativity (Atwood and Pretz, 2016; Pretz and Kuczma, 2025). Demographic diversity must be included in this work as prior research has shown that creativity measures reflect strengths especially among minoritized groups (Kaufman, 2010).

The creativity-infused course consisted of several components, making it difficult to know which aspects of the intervention were most responsible for the benefits seen (e.g., clarifying the nature of creativity, valuing creativity, practicing creative thinking, or providing feedback on creative work). Future work should help to discern what infusion is essential, comparing student creativity between courses that are focused more fully on the topic of creativity as well as others that include only one or two opportunities for feedback on creative work. In this study, creativity was implemented via individual and group activities, but future work should more carefully evaluate whether group projects are sufficient to convey the value of creativity and feedback on student success on these assignments. The reliance on group work may have diluted the impact of the intervention.

Although it was encouraging that the intervention increased creative self-efficacy and growth mindset in this small sample over one semester, it was disappointing that actual scores on the creative paper project were not higher than those in the traditional course. This may have been due to a ceiling effect as class averages in both sections were quite high. In the future, grading of creative work should be more critical. Another concern was the lukewarm student response about the usefulness of the feedback they received on their creative work. The study showed that the intervention had many of the desired effects, and the literature suggests that feedback is a key component of this benefit. Future work should explore student response to feedback in order to improve its effectiveness.

In the future, researchers should integrate reflection, empathy, and ethics into their creativity assignments. Desmet and Sternberg (2024 have advocated for the development of transformational creativity, “creativity that centers on the intention to effect a positive, meaningful, and lasting change in the world, one that not only generates novel and useful ideas but that also takes serious the responsibility for the moral impact of those ideas” (p. 2). In addition to an environment that supports risk-taking, provides scaffolded activities, and clear feedback via rubrics, they emphasize the importance of ethical considerations during the development of creative ideas, ensuring that innovation will serve the common good.

## 7 Conclusion

This pilot study responds to Beghetto's (2017) call for case studies in how to teach about, for, and with creativity, providing best practices and concrete examples of activities and assignments that can be integrated into any course and demonstrating preliminary evidence of their effectiveness in increasing student creativity. Creativity is essential for students in the 21^st^ century, and institutions of higher education should explore ways to foster students' ability to adapt to change and create solutions to address the complex problems in a rapidly changing environment.

## Data Availability

The raw data supporting the conclusions of this article will be made available by the authors, without undue reservation.
