# Supplementary material for: Infusing creativity into a college course: a pilot study
Source: Front Psychol. 2026 May 14;17:1768412. doi: 10.3389/fpsyg.2026.1768412 (PMC13216206; doi:10.3389/fpsyg.2026.1768412)
Supplement: Supplementary file 1 [file Supplementary_file_1.pdf]

Appendix

Figure 1

Minute Meme: Takeaway from class on effectiveness of various memory strategies

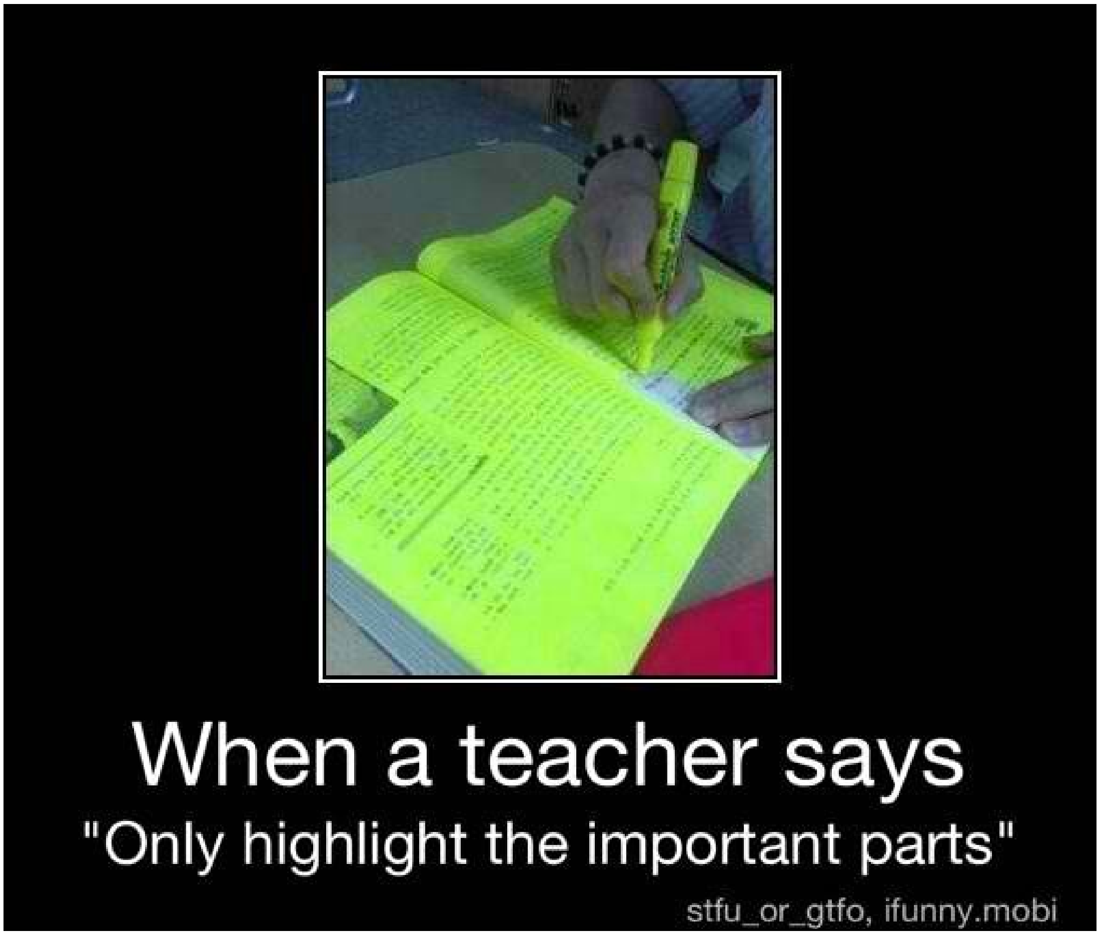

Figure 2

Innovative Illustration: Comic demonstrating factors affecting accuracy of eyewitness testimony

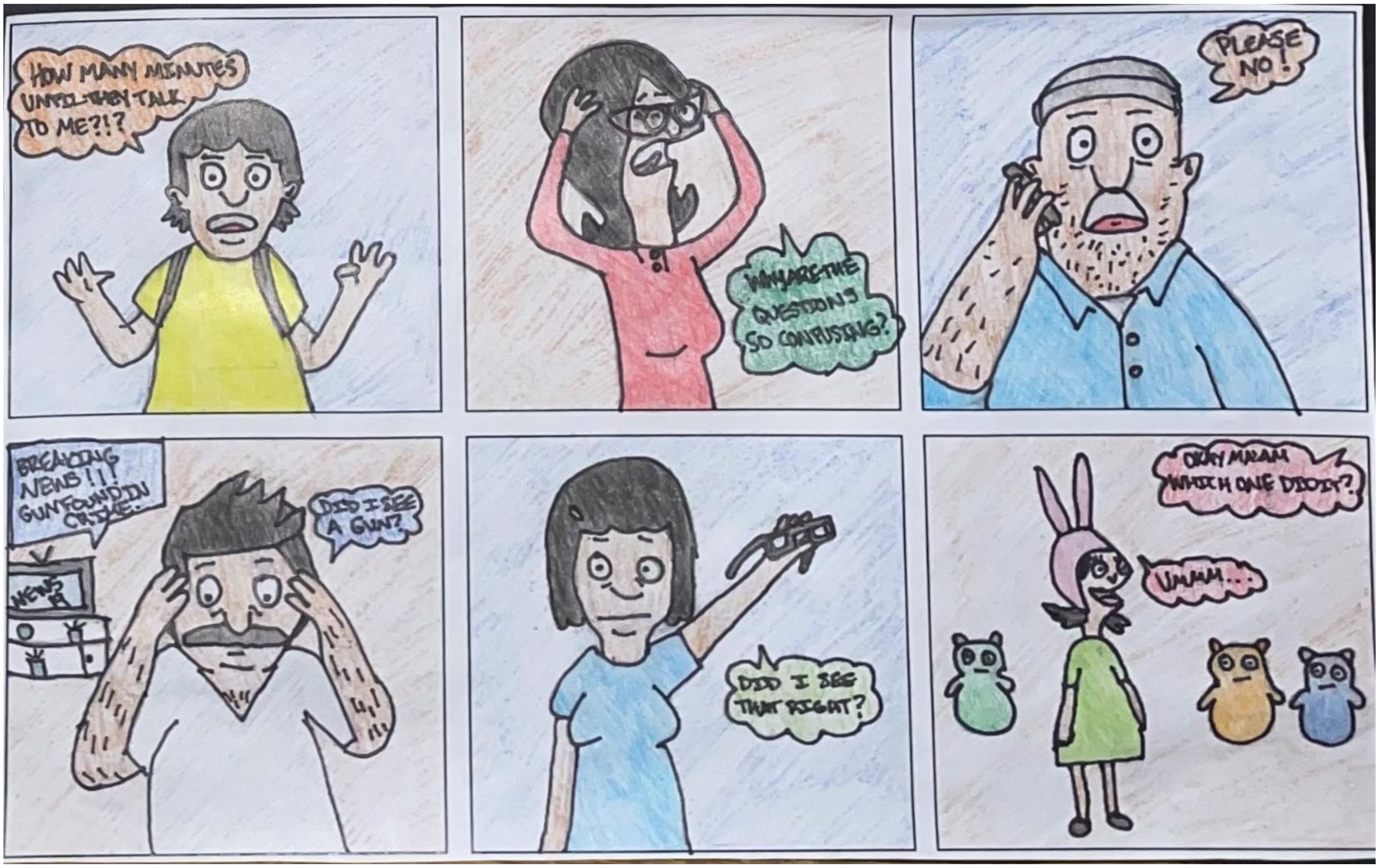

Note: Panel 1: How many minutes until they talk to me?!? Panel 2: Why are the questions so confusing? Panel 3: Please no! 4. Television shows Breaking news!!! Gun found in crime. Witness responds Did I see a gun? Panel 5: Did I see that right? Panel 6: Interrogator asks Ok ma’am, which one did it? She responds Ummm…

Figure 3

Creative Correction: Podcast evaluating and correcting representation of memory in the movie *The Hangover*

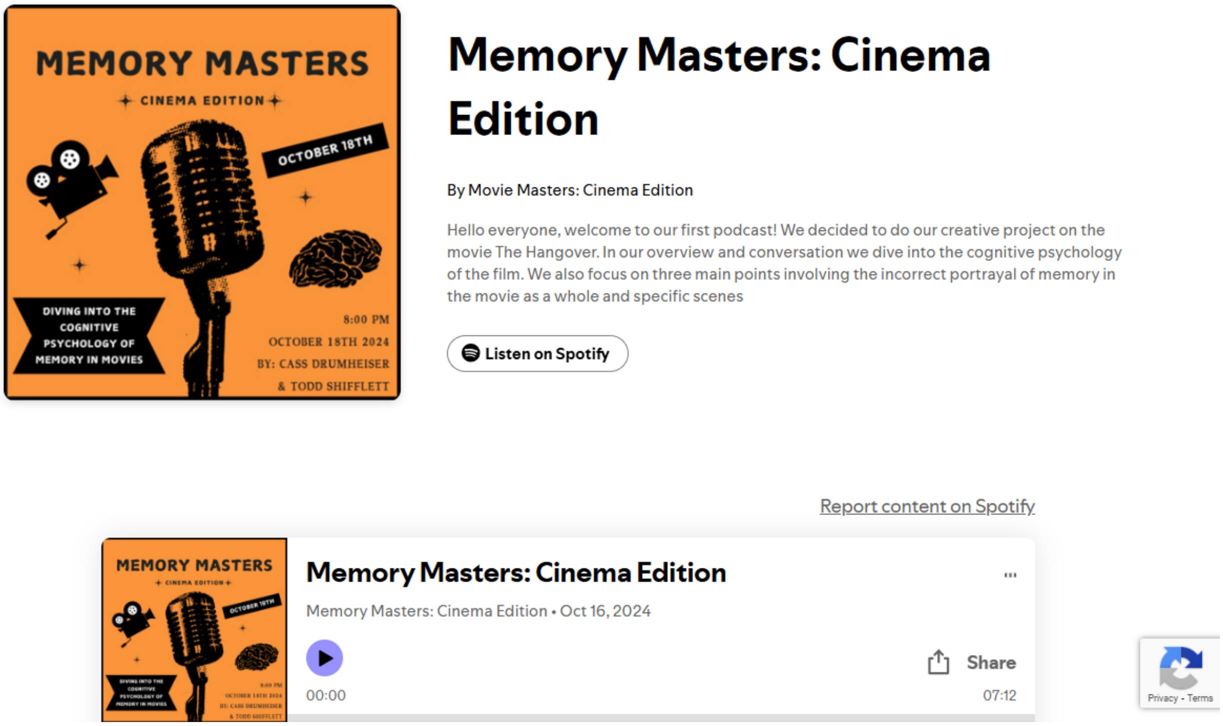

Figure 4

Creative Project Paper: Fashion design for a hoodie conveying gender differences in ADHD

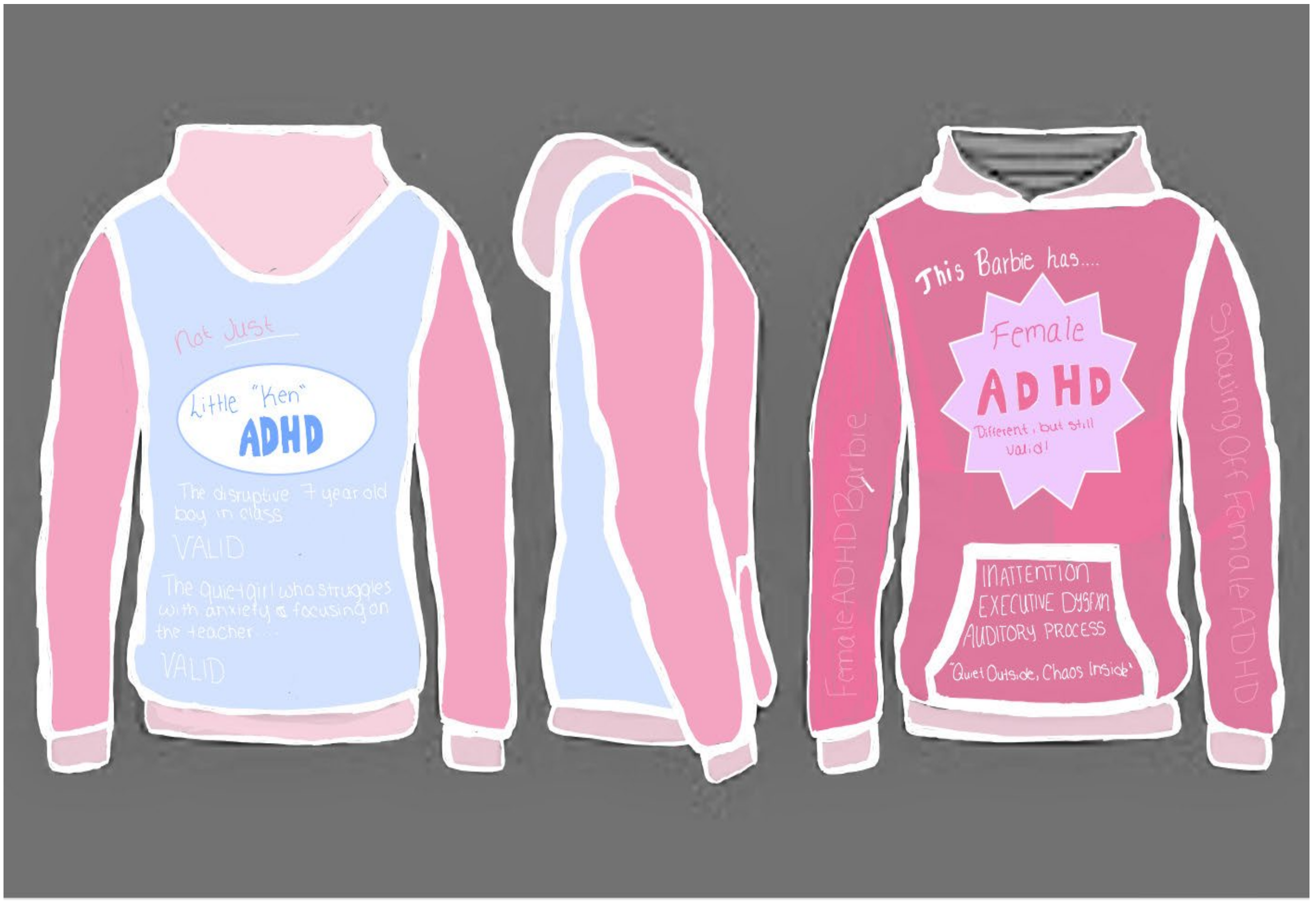

Note: Text on back of hoodie: Not Just Little “Ken” ADHD. The disruptive 7 year old boy in class: VALID. The quiet girl who struggles with anxiety and focusing on the teacher. VALID

Text on front of hoodie: This Barbie has Female ADHD. Different, but still valid. Inattention, executive dysfunction, auditory processes. “Quiet outside, chaos inside”

Text on sleeves of hoodie: Female ADHD Barbie. Showing off Female ADHD.
